# Post-vitrectomy delayed retinal breaks in proliferative diabetic retinopathy

**DOI:** 10.1186/s40942-023-00444-1

**Published:** 2023-02-01

**Authors:** Ramesh Venkatesh, Yash Parmar, Rubble Mangla, Shama Sharief, Naresh Kumar Yadav, Jay Chhablani

**Affiliations:** 1 Department of Retina and Vitreous, Narayana Nethralaya, #121/C, 1st R Block, Chord Road, Rajaji Nagar, Bengaluru, 560010 Karnataka India; 2 Medical Retina and Vitreoretinal Surgery, University of Pittsburgh School of Medicine, 203 Lothrop Street, Suite 800, Pittsburg, PA 15213 USA

**Keywords:** Proliferative diabetic retinopathy, Post-vitrectomy, Retinal breaks, Outcomes

## Abstract

**Purpose:**

To report a series of cases of post-operative new secondary retinal breaks following vitrectomy for proliferative diabetic retinopathy (PDR).

**Methods:**

This retrospective case series included data of patients diagnosed with post-operative retinal breaks following uneventful vitrectomy surgery for PDR from January 2018 to December 2021.

**Results:**

New post-vitrectomy retinal breaks in PDR were seen in 7% of eyes (n = 10/148 eyes; 10 patients). Age of study patients ranged from 45 to 69 years and there were 8 males. Vitreous surgery was performed for vitreous hemorrhage in six eyes, macular tractional retinal detachment in three eyes and epiretinal membrane in one eye. Tractional fibrovascular proliferation near the retinal break prior to its development was noted either pre- or intra-operatively in 8 eyes. Mean time interval between the vitreous surgery and secondary retinal break development was 6.4 months. Residual fibrous tissue post-surgery adjacent to the break was noted in 4 cases. Sclerosed retinal vessel was noted in 4 eyes and associated inner retinal thinning or schisis in 5 eyes. No retinal detachment was noted in any case. Prophylactic barrage was done in 4 eyes. Last follow-up interval ranged from 4 to 53 months and visual acuity ranged from 6/6 to 6/60. No subretinal fluid, traction or break enlargement was noted at the last visit.

**Conclusion:**

Delayed post-operative retinal breaks following vitrectomy are uncommon in PDR eyes. Careful preoperative evaluation of the retinal proliferations, intraoperative dissection of the membranes and regular post-operative reviews are vital in anticipating the development of delayed post-vitrectomy retinal breaks. Observation could be the management strategy for these breaks.

## Introduction

Proliferative diabetic retinopathy (PDR) leads to severe vision threatening complications such as vitreous haemorrhage (VH), tractional retinal detachment (TRD) and combined retinal detachment (RD) that may require surgical intervention in the form of pars plana vitrectomy [1]. The goals of vitrectomy in diabetic retinopathy include clearing media opacities, releasing antero-posterior and tangential vitreoretinal tractions, peeling of epiretinal membranes, endolaser photocoagulation to the ischemic retina in order to downregulate the vascular endothelial growth factor production and tamponade of retinal breaks [1]. Iatrogenic retinal breaks and hemorrhages are the most frequent intraoperative complications noted during the surgery [2–4]. These breaks occur in the areas of thin/atrophic retina during membrane peeling close to the retinal surface and have to be lasered all around. Prolonged surgical time and use of bimanual surgeries for complex TRDs can also lead to development of iatrogenic retinal breaks and rhegmatogenous RD in patients who have undergone vitrectomy for advanced diabetic eye disease [5].

Post-operative complications seen after diabetic vitrectomy include cataract, recurrent vitreous hemorrhage, rubeosis iridis and neovascular glaucoma [6–10]. Development of post-vitrectomy new retinal breaks in proliferative diabetic eye disease are not frequently encountered or reported in literature to the best of our knowledge. The mechanisms pertaining to the development of these new retinal breaks after vitrectomy needs to be elucidated. Anatomical and functional outcomes of such cases need to be reported to understand the severity of complications which can arise from these post-operative retinal breaks.

Herein, we intend to report a series of cases of post-operative new secondary retinal breaks following vitrectomy for PDR and also explain the possible pathogenesis and outcomes.

## Methods

In this retrospective case series, we collected and analysed the data of patients diagnosed with post-operative full-thickness retinal breaks following uneventful vitrectomy surgery for PDR from January 2018 to December 2021. The study complied with the tenets of the Declaration of Helsinki and was approved by the local Institutional Review Board/Ethics Committee. Because the study was a retrospective analysis, waiver for informed consent was obtained.

The primary inclusion criterion for this study was the identification of new full-thickness retinal breaks following vitrectomy for PDR performed by the single surgeon (RV). Cases of combined RDs and cases with iatrogenic retinal breaks noted intraoperatively during the surgical procedure were excluded. Data entry and analysis was done using the Microsoft® Excel 2019 version. The routine surgical steps followed in all PDR cases included: Anterior and core vitrectomy via the pars plana route using the 25-gauge microincision vitrectomy system, peripheral truncation of the vitreous in cases with posterior hyaloid separation or induction of posterior vitreous detachment where the posterior hyaloid was not separated, membrane peeling using the segmentation and/or delamination technique with the use of vitrectomy cutter, forceps or scissors, endocautery to the active bleeding points and use of endotamponade either with expansile gas or silicone oil in case of iatrogenic retinal breaks. In difficult cases of membrane peeling bimanual technique of membrane peeling was employed. The data collection included demographic details, indication for vitrectomy in PDR, vitreoretinal interface changes at the site of the existing retinal break prior to and after its development, surgical steps performed, time interval between the surgery and development of retinal break, characteristics of retinal break such as number and location, presence of adjacent sclerosed vessel, presence of inner retinal thinning or schisis around the retinal break on optical coherence tomography (OCT), development of RD, treatment provided to the retinal break, visual acuity prior to surgery, at the time of retinal break identification and at last follow-up visits and total follow-up interval.

## Results

**Fig. 1:**
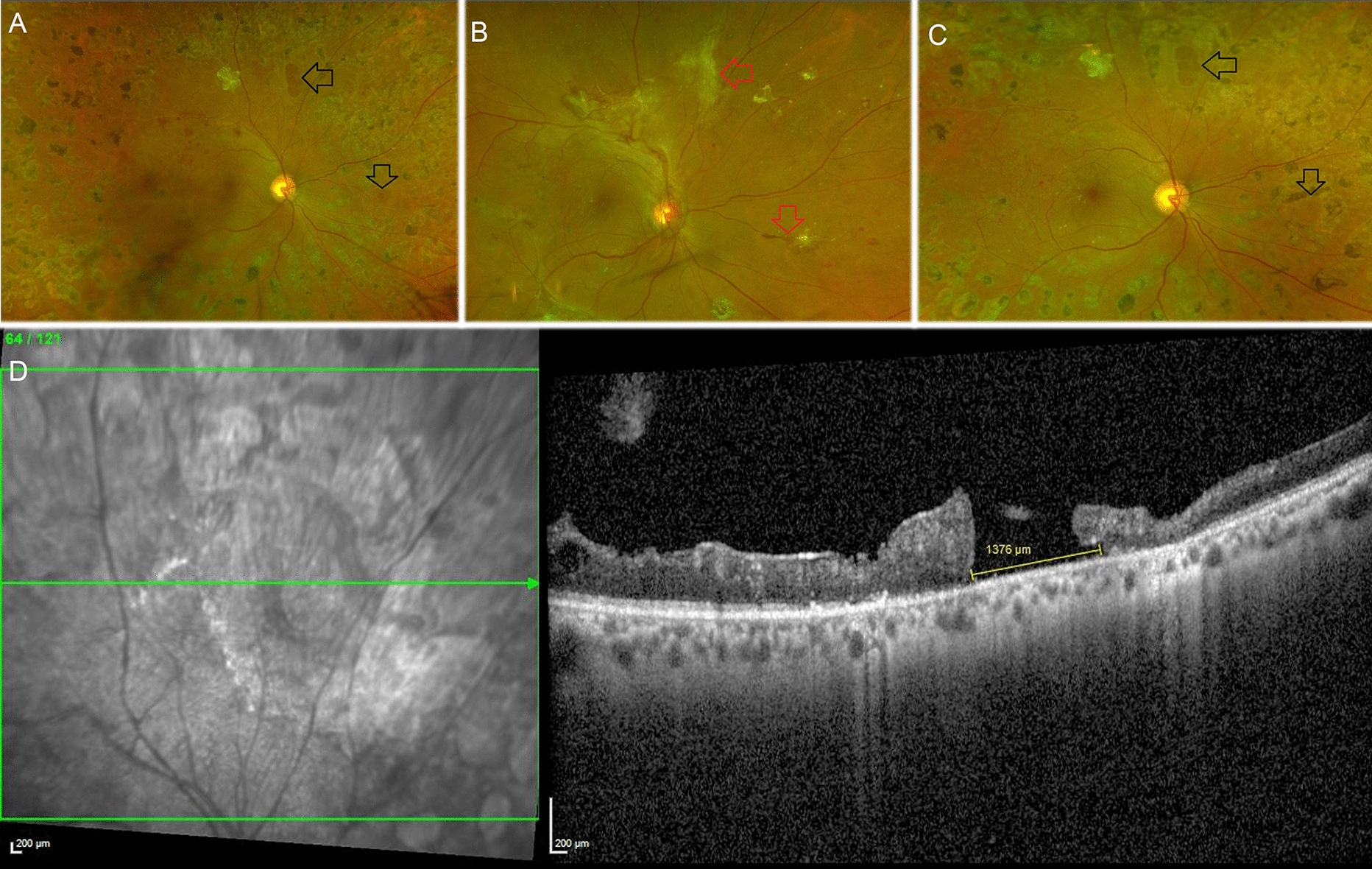
Details of case 3. **A**: A 46-year-old man with type 2 diabetes mellitus who was treated with oral hypoglycemic agents as well as injectable insulin developed multiple post-vitrectomy retinal breaks in the right eye three months after surgery for vitreous haemorrhage secondary to proliferative diabetic retinopathy. These retinal breaks were two in number (one superior to the optic disc and one nasal to it) and were located posterior to the equator (black arrows). **B**: Before the development of vitreous haemorrhage, a preoperative fundus image of the right eye shows the presence of dense fibrous proliferation at the sites where the retinal breaks eventually developed following surgery (red arrows). These retinal breaks were treated prophylactically with a barrage laser. **C**: The retinal breaks were well covered with laser scars at the last follow-up visit, 25 months after the surgery, and there was no new subretinal fluid or traction at the site of the retinal break (black arrows). **D**: Optical coherence tomography (OCT) scan through the superior post vitrectomy retinal break shows a large atrophic retinal hole measuring 1376 µm at the basal diameter with no overlying traction. The retinal layers surrounding the retinal break were disorganised, as seen on the OCT

**Fig. 2:**
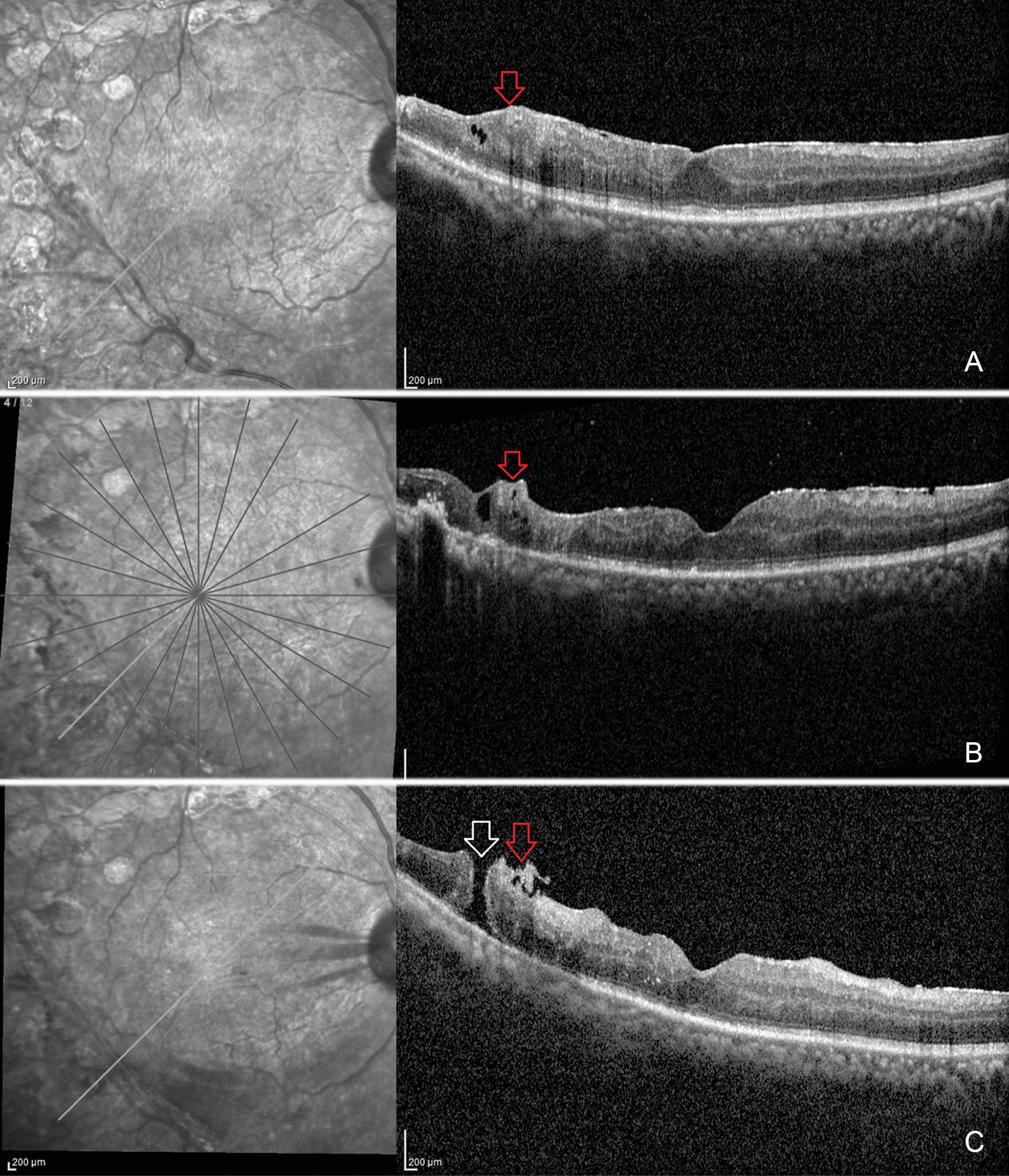
Sequential optical coherence tomography (OCT) scans demonstrating the possible mechanism of secondary post-vitrectomy retinal break. **a**: Preoperative OCT scan of the right eye fundus showing the epiretinal membrane (red arrow) extending right till the inferior arcade in a 57-year-old-male, previously operated for vitreous hemorrhage following proliferative diabetic retinopathy. Subsequently, patient redevelops vitreous hemorrhage for which he undergoes re-vitrectomy and removal of epiretinal membrane up to the retinal arcades. 2**b**: A residual preretinal membrane (red arrow) is noted along the inferotemporal arcade in the oblique line OCT scan done 1 month after the vitreous surgery. The inner retinal layers at that site appear disorganised with loss of retinal layer stratification and development of intraretinal cystic space. **C**: There is contraction of the preretinal membrane (red arrow) at the inferotemporal arcade eventually leading to development of full-thickness post-vitrectomy retinal break (white arrow) after 4 months post re-vitrectomy. There was no subretinal fluid surrounding the retinal break

**Fig. 3:**
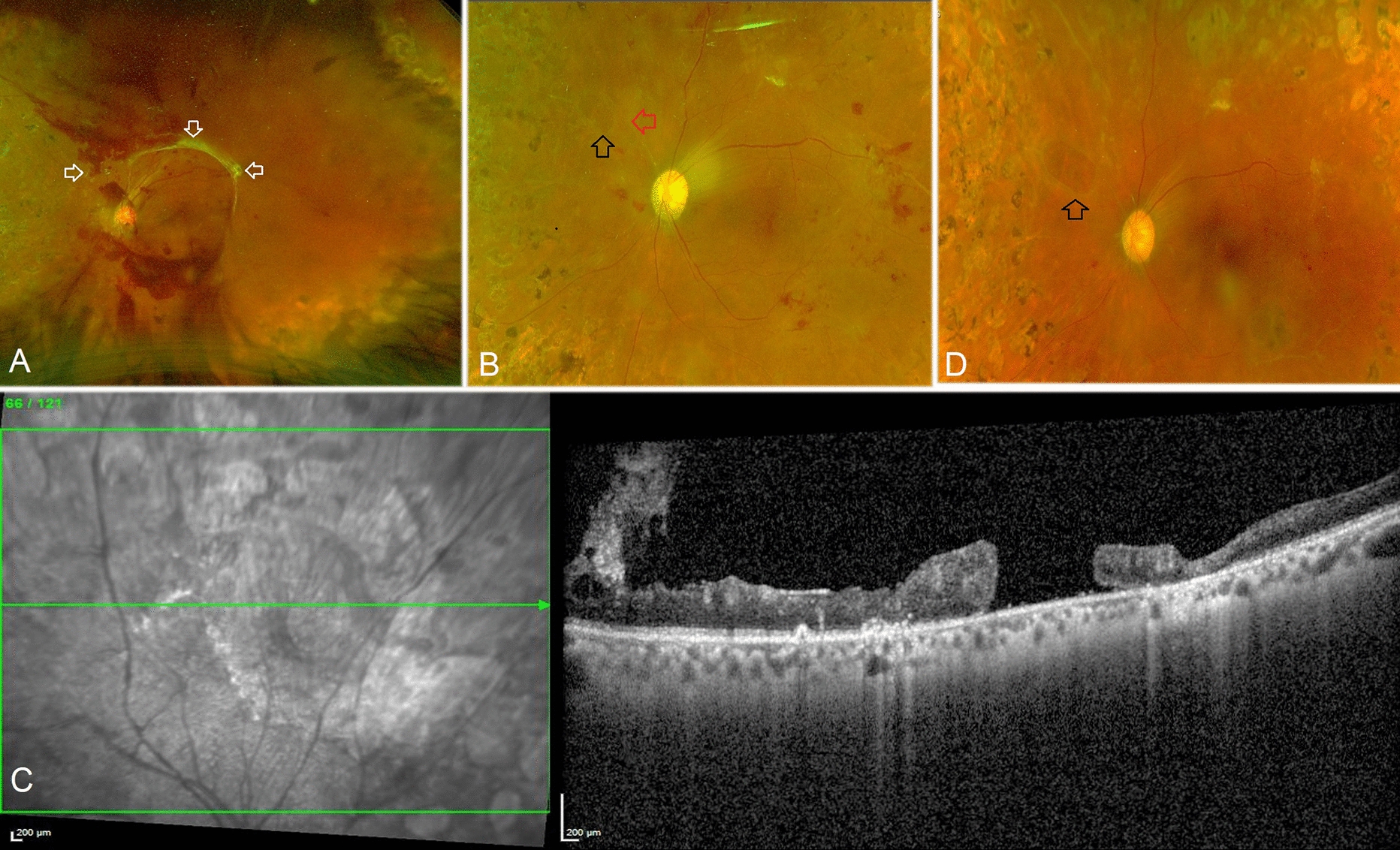
Details of case 4.  **A**: A 46 year-old female with type 2 diabetes mellitus for 15 years showed extramacular proliferations (white arrows) and preretinal heme over the posterior pole, with visual acuity lowering to 6/36. Following vitrectomy and membrane peeling with 20% SF6 endotamponade in the left eye, the patient's visual acuity improved to 6/6 at the 6 week post-operative visit. **B**: An oval full-thickness retinal break along the superonasal arcade posterior to the equator is observed 13 months after primary vitreous surgery (black arrow). Sclerosed superonasal retinal vessel (red arrow). **C**: An OCT scan of the retinal break revealed a large atrophic full-thickness retinal hole with no overlying traction. The retinal layers surrounding the retinal break were disorganized, as seen on the OCT. No prophylactic laser barrage was done surrounding the retinal break. **D**: At the last follow-up visit, 20 months after the primary vitrectomy surgery, there was no change in the retinal break (red arrow), and no new retinal breaks had developed. There was no development of retinal traction or subretinal fluid around the retinal break

For this study, case records of 198 eyes of 175 patients who underwent pars plana vitrectomy for different indications in PDR eyes were analysed. Cases with combined RDs were excluded from the study analysis. Mean age of the patients who underwent surgery was 56.23 ± 9.12 years. There were 140 males and 35 females who underwent surgery during this period. The main indications for which surgery was performed included VH (n = 110, 55%), epiretinal membrane removal (n = 15, 8%) and fovea involving or fovea encroaching TRD (n = 73, 37%). Iatrogenic intraoperative retinal breaks were noted in 20 (10%) eyes and were managed accordingly with endolaser and endotamponade. These eyes were excluded from further analysis. In remaining 178 eyes, no intraoperative complications occurred. Follow-up, post-operative details (at least ≥ 1 month) were available for 148 of the 178 (83%) eyes who underwent uncomplicated vitrectomy surgery for PDR. The retina remained attached in all 148 eyes at the last post-operative visit. During this post-operative period, we identified 10 (7%) eyes of 10 patients with type 2 diabetes mellitus and new post-vitrectomy retinal breaks in PDR. Case descriptions of these individual cases are summarized in Table 1. Age of study patients ranged from 45 to 69 years and there were 8 males in the study. The right eye was involved in six cases. The indication for vitreous surgery in these patients with PDR was VH in six eyes, TRD involving the macula in three eyes and presence of epiretinal membrane with retinal traction in one eye respectively. Presence of tractional fibrovascular proliferation at the site of the retinal break prior to its development was noted either preoperatively or intraoperatively in 8 of the 10 eyes. The key surgical steps performed in each case is mentioned in Table 1. The mean time interval between the vitreous surgery performed and identification of secondary retinal break was 6.4 months ranging from 3 to 15 months. The hole characteristics such as number and location are mentioned in detail in Table 1**.** Presence of residual fibrous tissue post-surgery adjacent to the retinal break was noted in 4 of the 10 cases. Presence of sclerosed retinal vessel in the area adjacent to the retinal break was noted in 4 eyes and associated inner retinal thinning or schisis was seen on OCT in 5 eyes. In 3 cases, OCT scan at the site of retinal break was not available. No RD was noted in any case in the presence of retinal break. Prophylactic barrage was done in 4 eyes while in the remaining 6 cases, the retinal breaks were not treated and were followed at regular intervals. The final follow-up interval ranged from 4 to 53 months in the study. The visual acuity at the last visit ranged from 6/6 to 6/60 in the study. No subretinal fluid, traction or enlargement of the retinal break was noted in any case even at the last follow-up visit. Clinical findings related to the cases is shown in Figs. 1, 2, 3

**Table 1:** Demographic, clinical and imaging features of patients with post-vitrectomy retinal breaks in proliferative diabetic retinopathy:

No	Age	Sex	Eye	Indication for surgery	Pre op VA	Membrane at the site of retinal break prior to surgery	Surgery done	Dye	ILM Peel	Tamponade	VA after surgery at the time of development of retinal break	Time interval for the development of retinal break (months)	No. of retinal breaks	Location: Anterior or posterior to equator	Quadrant of retinal break	Presence of overlying fibrous tissue	Presence of sclerosed vessel	Presence of retinal thinning	Prophylactic laser to the retinal break	VA last visit	Last follow-up duration (months)
1	57	M	RE	VH	HM	Yes	VIT + ERMP + EL + FAE	No	No	Air	6/12	4	1	P	Inferotemporal	Yes	No	Yes	Yes	6/12	13
2	55	M	LE	VH	CFCF	Yes	VIT + MP + FAE + EL + GAS	No	No	SF6	6/18	4	1	P	Superotemporal	No	No	Not assessed	No	6/6	41
3	46	M	RE	VH	CF @ 2 M	Yes	VIT + MP + EL + FAE	No	No	AIR	6/9	3	2	P	Superior, Nasal	No	No	Yes	Yes	6/9	25
4	45	F	LE	VH	6/36	Yes	VIT + MP + FAE + EL + GAS	No	No	SF6	6/6	13	1	P	Nasal	No	Yes	Yes	No	6/6	20
5	53	F	RE	Macular TRD	6/24	Yes	VIT + MP + FAE + EL + GAS	No	No	C3F8	6/60	2	5	P	Macula, superotemporal, superior, inferonasal, inferior	Yes	No	Yes	No	6/60	4
6	49	M	LE	Macular TRD	6/18	Yes	VIT + MP + ILMP + FAE + EL + GAS	BBG	Yes	C3F8	6/12	7	4	P	Superior, Nasal	Yes	Yes	Not assessed	No	6/12	8
7	63	M	RE	VH	HM	No	VIT + MP + EL + FAE	No	No	Air	6/9	3	1	P	Temporal	No	No	No	No	6/9	6
8	69	M	LE	ERM with retinal traction	6/9	No	VIT + ERMP + ILMP + EL + FAE	BBG	Yes	Air	6/9	10	1	P	Superotemporal	No	No	No	No	6/9	4
9	55	M	RE	VH	6/36	Yes	VIT + MP + FAE + EL + SO	No	No	SO	6/18	3	1	P	Superotemporal	Yes	Yes	Not assessed	Yes	6/18	10
10	61	M	RE	Macular TRD	CF @ 2 M	Yes	VIT + MP + FAE + EL + SO	No	No	SO	6/36	15	3	P	Temporal, superotemporal	No	Yes	Yes	Yes	6/12	53

*M* male, *F* female, *RE* right eye, *LE* left eye, *VH* vitreous hemorrhage, *TRD* tractional retinal detachment, *ERM* epiretinal membrane, *HM* hand motions, *CFCF* counting fingers close to face, *VIT* vitrectomy, *MP* membrane peeling, *FAE* fluid-air exchange, *EL* endo laser, *SO*-silicone oil, *ILMP* internal limiting membrane peeling, *ERMP* epiretinal membrane peeling, *SF6* sulfur hexafluoride, *C3F8* perfluoroproprane; *BBG* brilliant blue G

## Discussion

In summary, this series identifies cases of post-vitrectomy new retinal breaks in patients with advanced proliferative diabetic eye disease. These retinal breaks were seen in 7% of cases operated with PPV for various indications in PDR eyes and were spotted after a mean interval of 6 months following the vitreous surgery, were round or oval in configuration, single or multiple in number, located posterior to the equator and did not lead to RD.

Several assumptions could be provided for the development of these retinal breaks after vitrectomy in PDR eyes. Damage to the retinal layers following diabetic vitrectomy surgery can occur due to surgical trauma while vitrectomy or internal limiting membrane (ILM) peeling [11], toxicity due to the use of vital dyes like Brilliant blue G (BBG) [12, 13], phototoxicity due to the excessive use of endoilluminator light very close to the retinal surface for prolonged periods [14], excessive use of endocautery at high intensity and toxicity due to the inappropriate antibiotic dose in the saline infusion. In this series, no intraoperative iatrogenic retinal breaks were noted and antibiotics were not added to the infusion solution.

In eyes with PDR, there is extensive deep capillary plexus loss which causes disorganisation of the retinal inner layers and structural disintegrity [15]. The retinal neovascularisation arises from the abnormal deep capillary plexus at the junction of the perfused and non-perfused areas [16]. The persistent traction by the posterior cortical vitreous on the retinal neovascularisation and subsequently on the inner retinal layers causes further inner retinal weakening [17]. In 8 of the 10 cases in our series, we found the presence of a pre-existing overlying fibrous proliferation pre-operatively at the site where the retinal break eventually developed. In one case (case 7), the preoperative fundus was not visualised due to dense VH while the intraoperative fundus after clearing the VH showed a fibrous proliferation at the site where the post-operative retinal break developed. In 4 of the 8 eyes, the pre-existing fibrous proliferation was completely removed and no intraoperative retinal breaks were identified at those sites. In the remaining 4 cases, the residual stump of the proliferation was retained. Thus, this clinical finding supports our assumption, that the antero-posterior and tangential tractions on the retinal proliferation or clinically invisible retinal microbreaks could play an important role in the formation of post-vitrectomy retinal break. Also, one needs to be extra cautious during surgery not to completely detach the proliferation from its base as it can cause further inner retinal schitic changes, leading to post-vitrectomy retinal break formation. Endo diathermy of the bleeding vascular proliferation with high intensity power could also damage the retinal layers further leading to full-thickness retinal breaks later [18]. Thus, during surgery, adequate diathermy of the overlying bleeding retinal proliferations at a minimally sufficient power should be considered to prevent secondary retinal break development.

In 2 cases of the current series, ILM peeling over the posterior pole was done after staining with BBG. The role of ILM peeling in vitreous surgery for PDR complications has been questionable. While a fewer cases of recurrent epiretinal membranes and macular edema are noted in eyes with ILM peeling, the visual acuity gain does not significantly differ from the cohort of patients who did not undergo ILM peeling [19, 20]. ILM peeling causes inner retinal layer damage and weakening and can lead to development of post operative retinal breaks in eyes with thin and atrophic retina [21]. In both cases, the retinal breaks developed at sites away from the peeled ILM area. The contraction of the residual stiff ILM at the junction of the peeled inner and non-peeled outer regions could be responsible for the development of these post-vitrectomy retinal breaks as described by Brouzas et al.[22] Thus, ILM peeling in eyes with macular TRD or primary epiretinal membrane removal needs to be performed only when essential.

In vitrectomised eyes with PDR, development of sclerosed vessels leads to progressive increase in the areas of retinal ischemia. The resultant structural changes in the inner retinal layers assisted by the tangential contraction of the residual fibrous proliferation and adjacent ILM can lead to the development of secondary retinal breaks. Hence, regular follow-up of vitrectomised eyes with PDR is vital for the identification of such post-operative retinal breaks.

A few other typical features noted in this case series were that all the retinal breaks were noted posterior to the equator, where the laser scars were deficient and none of the cases were complicated with the development of rhegmatogenous RD during the mean follow-up of 18.4 months. In this series, prophylactic laser barrage to the retinal break did not seem to be protective from developing RD, as no RD was noted even in eyes who did not undergo prophylactic barrage laser to the retinal breaks. These observations further strengthen the belief that post-vitrectomised retinal breaks in eyes with PDR are atrophic retinal breaks, not caused by dynamic antero-posterior or tangential contraction forces.

The small number of cases is the main drawback of our study. Nonetheless, in our study we report a novel finding of delayed secondary retinal breaks in post-vitrectomised eyes with PDR, furnish its possible pathogenesis and portray its outcome over a long follow-up period. To the best of our knowledge, this phenomenon of delayed secondary retinal breaks in post-vitrectomised eyes with PDR has not been reported in literature so far.

In conclusion, careful preoperative assessment of the retinal proliferations and areas of retinal thinning, intraoperative dissection of the membranes and regular post-operative reviews are vital in anticipating the development of delayed secondary atrophic retinal breaks in post-vitrectomised PDR eyes. Observation with no intervention could be the management strategy for these breaks with no risk of development of RD.

## Data Availability

The datasets used and/or analysed during the current study are available from the corresponding author on reasonable request.

## Abbreviations

PDR: Proliferative diabetic retinopathy
VH: Vitreous hemorrhage
TRD: Tractional retinal detachment
RD: Retinal detachment
OCT: Optical coherence tomography
ILM: Internal limiting membrane
BBG: Brilliant blue G
